# A lightweight weak semantic framework for cinematographic shot classification

**DOI:** 10.1038/s41598-023-43281-w

**Published:** 2023-09-26

**Authors:** Yuzhi Li, Tianfeng Lu, Feng Tian

**Affiliations:** Shanghai Film Academy, Shanghai University, Shanghai, 200072 China

**Keywords:** Computer science, Scientific data

## Abstract

*Shot* is one of the fundamental unit in the content structure of a film, which can provide insights into the film-director’s ideas. By analyzing the properties and types of shots, we can gain a better understanding of a film’s visual language. In this paper, we delve deeply into the task of shot type classification, proposing that utilizing multimodal video inputs can effectively improve the accuracy of the task, and that shot type classification is closely related to low-level spatiotemporal semantic features. To this end, we propose a Lightweight Weak Semantic Relevance Framework (LWSRNet) for classifying cinematographic shot types. Our framework comprises two modules: a Linear Modalities Fusion module (LMF Module) capable of fusing an arbitrary number of video modalities, and a Weak Semantic 3D-CNN based Feature Extraction Backbone (WSFE Module) for classifying shot movement and scale, respectively. Moreover, to support practical cinematographic analysis, we collect FullShots, a large film shot dataset containing 27K shots from 19 movies with professionally annotations for movement and scale information. Following experimental results validate the correctness of our proposed hypotheses, while our framework also outperforms previous methods in terms of accuracy with fewer parameters and computations, on both FullShots and MovieShots datasets. Our code is available at (https://github.com/litchiar/ShotClassification).

## Introduction

Video data is an integral component of the modern internet and a key research area in computer vision. With machine learning and deep learning technologies, videos can be automatically recognized and classified, allowing video websites to autonomously review them. Comparing to user-uploaded videos, films are produced by professional directors after editing and adding post-production effects, which means have a longer duration and higher resolutions. Thus, research on films appears to be relatively scarce compared to user-uploaded videos and other genres.

Video classification is a common task in computer vision, including action recognition^1,2^, micro-video classification^3,4^, video emotion classification^5^, etc. These tasks identify target categories by extracting high-level spatio-temporal semantic information from video data^6^. Specifically, action recognition analyzes objects or activities in videos to associate with specific actions; micro-video classification examines video themes uploaded by users; video emotion classification determines human emotions based on facial expressions, body movements, and poses. We find that videos in these tasks are often 5–30 s long, allowing direct use of the full video as algorithm input. However, in movie analysis, a film may last several hours, making it difficult for artificial intelligence to analyze the entire movie. Instead, shot segmentation^7,8^ or scene segmentation^9^ algorithms are typically used to divide movies into thousands of shots or dozens of scenes. At the scene level, analysis often focuses on identifying characters^10,11^ , while at the shot level we examine here, intrinsic attributes like shot movement and shot scale are analyzed, which are collectively referred to as **Shot Type Classification**.

Over the past decade, there has been relatively limited research on shot type classification, which we suggest stems from two primary reasons: (1) Lack of practical application needs: Most videos on video websites are edited and spliced together from multiple shots by users. For video websites, when using artificial intelligence to enhance the efficiency of content review, it is only necessary to focus on the content information of the video without the need to recognize the shot attributes of the video. (2) Lack of benchmark datasets: Compared to ordinary user-uploaded videos, movies as a form of art with greater investment and longer production cycles have more stringent copyright protections, this impedes the construction of benchmark datasets containing full-length movie shots. Existing movie shot datasets sidestep legal hurdles but have limitations: MovieNet^12^, the first general-purpose movie analysis dataset, provides only 3 frames per shot; MovieShots samples selected clips from thousands of movie trailers; CineScale^13^ offers just the first frame per second based on movie duration.

Shot type classification holds considerable research value for two main reasons: (1) Shot material management: In large-scale photography databases or film shots, the number of shots taken by the camera is numerous, video producers need to spend a significant amount of time organizing shot material. Shot type recognition can automatically classify shot material, thereby facilitating post-production video editing work. (2) Film comprehension: In *Cinemetrics*^14^ theories, film style can be measured and analyzed systematically and digitally by analyzing elements such as shot duration, camera motion, and shot scale. In related research, shot attributes are manually annotated and statistically analyzed, which is relatively inefficient. However, deep learning methods for shot type classification can quickly identify the attributes of film shots, providing richer research samples for *Cinemetrics* studies.

To further study the shot type classification task, we construct a shot dataset comprising 27K shots from 19 films called **FullShots**. We remove content-irrelevant segments, such as opening, ending, and black screens, as well as a small number of shots with indeterminate shot types, such as shots with multiple movement types. Next, we annotated each shot clip with scale and movement labels. Compared to **MovieShots**^15^, we make two major improvements: (1) Guided by film theory proposed by Daniel Arijon in in *“Grammar of the film language”*^16^, we expand the shot movement categories from 4 to 8 and scale categories from 5 to 6 (see “Shot categories” section for details). (2) MovieShots only selects shots with notable objects or characters present, but some shots (e.g. *Long Shot*) may lack noticeable subjects, we posit these shots are equally important as *Subject-Centric* shots for shot classification task. Therefore, we annotate most shots across the entire film.

We then analyze previous research on shot type classification and discover that in many methods, in addition to frames, multiple additional video modalities have been used as extra inputs to the method, such as optical flow maps^15,17^, segmentation maps^18^, and saliency maps^19^. Although obtaining these modalities requires additional data preprocessing, they significantly improve the accuracy of shot type classification tasks.

Additionally, in video classification tasks such as action recognition, the shallow layers of neural networks extract low-level spatiotemporal semantic cues—local textures, shapes, edges—from individual video frames. Meanwhile, the deeper layers integrate these low-level features across broader spatiotemporal contexts to represent high-level semantic concepts—complete actions or activities. However, when analyzing shot types, humans first detect primary shapes and contours in each frame, then track their evolution along the temporal axis, disregarding specific objects or actions. This suggests a strategy for shot classification with deep learning: employ lightweight networks with fewer layers, or “weak semantic relevant” networks in our terminology. Compared to conventional deep networks, these streamlined architectures have drastically fewer parameters and are far less computationally intensive.

Based on the preceding analysis, we propose that shallow neural networks can enable more lightweight shot classification given the same video input modalities. To that end, we devised an architecture called the Lightweight Weak Semantic Relevance Network(LWSRNet). This architecture comprises two main modules: the first is the Linear Modalities Fusion Module (LMF Module), fuses inputs of various video modalities, and the Weak Semantic Feature Extraction Module (WSFE Module), which is responsible for extracting significant spatio-temporal features from fused multi-modal inputs. We then conduct various experiments on both the MovieShots and FullShots datasets to evaluate the effectiveness of our proposed method. The results show that our approach achieves better results with fewer parameters than previous methods in both datasets, and tasks on FullShots are more challenging than on MovieShots.

The following sections of our article are organized as follows: In “Related work” section, we provide an overview of relevant work in film analysis, shot movement classification and shot scale classification. In “LWSRNet: lightweight weak semantic relevance network” section, we introduce our proposed lightweight network architecture for shot type classification, and discuss the composition of the LMF Module and WSFE Module. In “FullShots Dataset” section, we introduce FullShots, a large film shot dataset from complete movies. In “Experiments” section, we conduct a series of experiments on MovieShots and FullShots, and provide several ablation studies to demonstrate the effectiveness of our proposed LWSRNet architecture. In “Conclusion” section, we conclude the entire article and suggest further research directions for cinematographic shot classification.

The contributions of this work are as follows: (1) We introduce FullShots, a dataset consisting of 27,000 shots selected from 19 movies, professionally annotated with shot movement and shot scale information. (2) We propose LWSRNet, a lightweight network architecture for recognizing shot types that can handle inputs from any number of video modalities. Following experiment results demonstrate that our proposed architecture achieves better performance than prior methods in the field of shot type classification while having fewer parameters and lower computational complexity. Notably, in the shot movement classification task, our proposed architecture significantly outperforms existing methods^15,17,20^ under equivalent input conditions.

## Related work

### Film analysis

As a prevalent form of video content, movie analysis has been the subject of numerous studies, including movie dataset construction^12,15,21^, movie scene recognition^22^, movie scene segmentation^9^, online person search^11,23^, movie character recognition^10,24^, movie segment synopsis^25^, and shot type classification^15,17,20^. In this paper, we perform both movie dataset construction and shot type classification tasks. We have drawn upon the construction methodology of prior works^13,15,21^ to propose FullShots, a dataset derived from complete films that more aptly addresses the practical requirements of cinematic shot analysis, then we propose a lightweight framework for cinematographic shot classification that is more efficient compared to previous methods.

### Shot movement classification

Traditional methods for recognizing camera motions in videos involve extracting manually designed features, such as the non-parametric motion descriptor CAMHID^26^ and the 2D motion histogram 2DMH^27^, which are then classified using support vector machines (SVM). In deep learning-based approaches, RO-TextCNN^17^ generates a one-dimensional angle histogram using the video’s optical flow information and extracts multi-scale image information with a TextCNN-Based structure. SGNet^15^ separates foreground and background from the video by using a subject generation module, which is then used as inputs to a residual-linked dual-stream network^28^.

Optical flow is generated by the movement of foreground objects or the camera in the video, in action recognition tasks, optical flow can eliminate background noise and other irrelevant features, at the same time, in the task of shot movement classification, optical flow can be used to directly analyze the direction of camera motion. In our proposed method, the optical flow map is also used as a video modality for recognizing camera motion types. However, since in film shots, characters and the camera may move simultaneously, only using optical flow as input for our framework may lead to ambiguity. Therefore, we use video frames and optical flow maps as input to the feature extraction backbone for shot movement classification, which directly learns the camera motion features. Moreover, we design the Movement Branch, a shortcut path providing feature vectors from original frames to improve framework performance.

### Shot scale classification

In the context of shot scale classification, traditional methods recognize shots from sports videos^29^ using low-level texture features. After the first application of convolutional neural networks for identifying shot scale categories in cinematography^18,30^ employ three parallel VGG16 networks to extract features concurrently from frames, semantic segmentation maps, and hypercolumns, then stacking learning techniques were used to improve accuracy. In^20^, a vertical and horizontal pooling method was proposed to handle video frames with different aspect ratios. In addition to video frames, segmentation maps and saliency maps^19^ are commonly used as input modalities for shot scale classification, which aim to divide the screen into various parts and highlight the main objects in the scene. Previous researches^18,30^ have focused on direct recognition for individual photographs, while in film shots, we assume that each shot has only one scale type. Furthermore, we find that visual texture features from frames are equivalently important for shot scale classification, to address this, we employ a pyramid downsampling block^31^ to extract features from origin frames. Specifically, we use a pre-trained ResNet50^32^ model as the pyramid backbone, and the parameters of this block will not be updated during the training stage.

## LWSRNet: lightweight weak semantic relevance network

**Figure 1:**
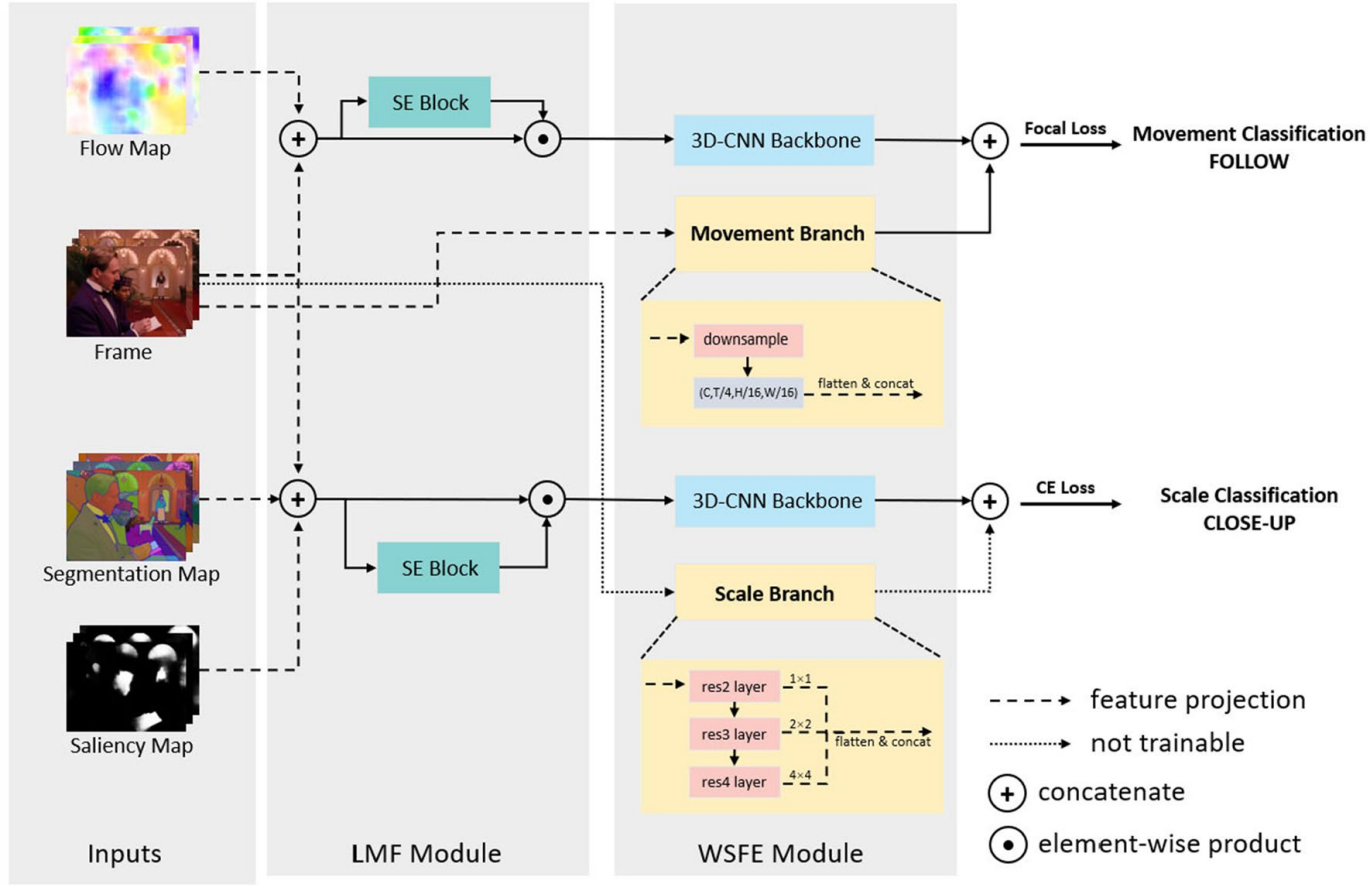
Overall architecture of our proposed LWSRNet. We use *Frame* and *Flow Map* for Cinematographic Movement Classification. *Frame*, *Segmentation Map* and *Saliency Map* for Cinematographic Scale Classification. In order to improve the effectiveness of the method, we design feature supplementation strategies for each of the two tasks, namely *Movement Branch* and *Scale Branch*.

In this section, we propose LWSRNet, a Lightweight Weak Semantic Relevance Network Architecture for cinematographic shot classification. The overall framework is illustrated in Fig. 1.

A shot is composed of a varying number of images, each of which is referred to as a frame. Since processing variable-length time-series data in a convolutional neural network is challenging and video data is typically redundant, we adopt the frame sampling approach proposed in TSN^33^. Specifically, We divide a shot into *N* segments, and randomly sample one frame from each segment. For MovieShots^15^ we set *N* as 8, and for our proposed FullShots, we set *N* as 16, due to the average shot duration is much longer.

In LWSRNet, we firsrtly suppose that the intrinsic attributes of shots (including movement and scale) are weakly correlated with high-level semantic information (e.g. character actions and object categories). Instead, shot types are more related to low-level spatio-temporal semantic information, such as time-varying texture information.Therefore, we suppress the learning of high-level semantic information by reducing the depth of the feature extraction network, thereby reducing the parameter and computational complexity of the framework. Additionally, we find that in previous methods^15,17,20,21^, only *Mean Function* or *Long Short Term Memory (LSTM)* structures have been used to aggregate temporal information after the feature extraction module. However, the first half of these methods still process individual frames in parallel, which is not conducive to capturing temporal information. Therefore, we choose a shallow C3D^2^ network as the backbone to effectively capture spatio-temporal features from shots, and then we propose information supplement strategies separately for shot movement classification (**Movement Branch**) and shot scale classification (**Scale Branch**).

In the next two subsections, we present the Linear Modality Fusion Module, which fuses multi-modal video inputs, and the Weak Semantic Feature Extraction Module, which performs feature extraction using the carefully designed Movement Branch and Scale Branch.

### Linear modalities fusion module (LMF module)

In computer vision, both *early fusion* and *late fusion* can improve the performance and generalization of algorithms. In terms of cinematographic shot classification methods,^15,18^ can be regarded as using a late fusion strategy to improve accuracy. However, the use of several parallel network architectures for late fusion increases the overall computational complexity, and do not allow for modality interaction during the process.

Drawing on our analysis from the previous section, in addition to shot frames, we use optical flow maps as additional input for shot movement classification, segmentation maps and saliency maps as additional inputs for shot scale classification. Therefore, our early fusion LMF Module needs to be able to accommodate any number of video modalities.

Below we describe the video modalities used in our framework and detail the acquisition method for each modality:

#### Frame

A sequence of images obtained by sampling the shots, denoted as $I_{frame}\in {\mathbb {R}} ^{C\times N\times H\times W}$ , where *C* represents the number of channels (usually 3), *N* represents the length of sequence, H and W represent the height and width of the shot, respectively.

#### Optical flow map

Optical flow is a vector field that describes the pixel motion between adjacent frames, representing the speed and direction of motion for each pixel. Following SGNet^15^, FlowNet 2.0 model pre-trained by FlyingChairs Dataset^34^ is used in our framework to extract the flow map from each shot clips. The optical flow map is represented as $I_{flow}\in {\mathbb {R}} ^{C\times N\times H\times W}$.

#### Segmentation map

The segmentation map is obtained by a semantic segmentation network, which labels each pixel in the image as a different semantic category. In shot type classification tasks, the segmentation map provides detailed control information about the frame to the feature extraction backbone. In this paper, we use a UNet^35^ framework trained on the VOC dataset as the semantic segmentation network, which directly segments $I_{frame}$, and the segmentation map is represented as $I_{seg}\in {\mathbb {R}} ^{C\times N\times H\times W}$.

#### Saliency map

The saliency map is obtained by the saliency detection methods, which indicates which regions in the frame are most attractive and important to human vision. In this paper, we use a pre-trained R3Net^36^ model trained on the MSRA10K dataset to extract the saliency map from $I_{frame}$. The saliency map is represented as $I_{saliency}\in {\mathbb {R}} ^{C\times N\times H\times W}$, particularly, the number of chanells in the saliency maps is one, which represents the degree of attention of each pixel by a digit ranges from 0 to 255.

In the LMF Module, each input video modality undergoes a linear 3D convolution layer (without nonlinear activation functions) for feature projection, after which they are directly concatenated. Then, to reduce the number of input parameters for the feature extraction module while retaining as many fused features as possible, an adaptive pooling layer is used to reduce the number of output channels of the LMF module to *D* (usually *D* is set to 64). Furthermore, we a Squeeze and Extraction Block^37^ is added before output in order to enable the framework to learn the channel weight assignment between different modalities from the input data. The LMF Module’s procedure can be formulated as Eq. (1), where $\phi _{m/s}$ denotes the output of LMF Module(*m* for movement and *s* for scale), $\alpha _{m/s}$ denotes the channel weighting factor, ${M}_{m/s}$ denotes the feature projection process.

1 $$\begin{aligned} \begin{aligned}\phi _{m} & = \alpha _{s} \odot {M}_{m} \left( I_{frame}\oplus I_{flow}\right) \\ \phi _{s} & = \alpha _{m} \odot {M}_{s} \left( I_{frame}\oplus I_{seg}\oplus I_{saliency}\right) \\ \end{aligned} \end{aligned}$$

### Weak semantic feature extraction module (WSFE module)

#### Shallow 3D-CNN backbone

Previous approaches^15,17,20^ for shot type classification typically aggregate temporal dimensions after feature extraction. In our framework, owing to the 3D convolutional networks has superior capability to more comprehensively model the relationships between spatial and temporal dimensions in contrast to traditional 2D convolutional networks^2^, we adopt a 3D convolutional backbone as the feature extractor module. However, directly using C3D backbone would significantly increase framework’s computational complexity. Therefore, considering the importance of low-level semantic features in shot classification tasks, we choose a *Depth-3 C3D* as the backbone. The backbone outputs a *D*-dimensional vector (usually *D* is set as 2048), which is then passed through two fully connected layers to predict the category vector. The process of feature extraction can be formulated as follows, where *N* denotes the feature extraction process, $\varphi _{m/s}$ denotes the output eigenvector:

2 $$\varphi _{m/s}= {N}(\phi _{m/s})$$

#### Movement branch

For shot movement classification, our framework uses $I_{frame}$ and $I_{flow}$ as input modalities. However, the flow variation reflects both character and camera movement, which can create ambiguity in some shots where both types of motion occur simultaneously, such as tracking shots. Therefore, we add a *Movement Branch* that enhances the movement classification. Specifically, $I_{frame}$ passes through an extra **non-linear** 3D convolution layer before undergoing an average pooling layer. The result is then flattened into a 1D vector, which is concatenated with the vector $\varphi _{m}$.

#### Scale branch

For shot scale classification, our framework uses $I_{frame}$, $I_{seg}$ as input modalities. In addition, we find that texture information from origin video frames is equally important, and assume that there is only one scale type in a single shot. Inspired by^31^, we introduce a Scale Branch for scale type classification. We use the middle sampled frame from $I_{frame}$ and pass it through a pre-trained ResNet50 model to output pyramid-level features, which are then averaged, pooled, and flattened into a 1D vector. This vector is concatenated with the output vector $\varphi _{s}$ from the backbone network.

The entire process of shot movement classification and shot scale classification can be formulated as Eq. (3).

3 $$\begin{aligned} \begin{aligned} p_{m/s}= {F}_{m/s}(\varphi _{m/s} \oplus \mu _{m/s}; \theta _{m/s}) \\ \end{aligned} \end{aligned}$$

where $\mu _{m/s}$ denotes the one-dimensional feature vectors obtain from the Movement Branch and the Scale Branch, ${F}_{m/s}$,$\theta _{m/s}$ represent the Classifier Layers with their parameters and $p_{m/s}$ denotes the predicted vector of our framework.

#### Loss function

We use cross-entropy loss as the classification loss for shot scale classification. For shot movement classification, static shots usually account for a large proportion of shot samples,to mitigate this imbalance, we employ focal loss^38^as the classification loss, with the $\alpha$ value of *Static* type set to 0.3 and the remaining 0.7 weight evenly distributed to the other categories. The loss functions are formulated as Eqs. (4) and (5), where $y_{i,m/s}$ and $p_{i,m/s}$ denotes the ground truth and the predicted vector of the *i*-th sample for movement/scale category.

4 $$\begin{aligned} \begin{aligned} L_s=-\sum _{i=0}^{K}y_{i,s}log(p_{i,s}) \\ \end{aligned} \end{aligned}$$

5 $$\begin{aligned} \begin{aligned} {L}_m=-\sum _{i=0}^{K}\alpha _{m}y_{i,m}(1-p_{i,m})^{\gamma }log(p_{i,m}) \\ \end{aligned} \end{aligned}$$

## FullShots dataset

Before introducing our dataset, we briefly discuss *MovieShots*^12^*(Available)* and its subsequent work *MovieShots2*^21^*(Not Available)*. MovieShots is currently the benchmark dataset for shot type classification, consisting of 46K shot clips obtained from 7,858 movie trailers, annotated with five scale categories (LS, FS, MS, CS, ECS) and four movement categories (static, motion, push, pull). On the other hand, MovieShots2 focuses on video scenes (a scene consists of several consecutive shots), comprising 15K shot samples and are annotated with movement, scale, camera position, and shot time boundaries.

We argue that shots from movies are diverse and not limited to the Subject Centric Lens mentioned in MovieShots^15^. Therefore, we propose the FullShots dataset, consisting of 27,000 movie shots from 19 complete movies, uniformly annotate with shot scale and movement labels.

### Shot categories

Regarding shot categories, shot movement and scale are the most commonly used attribute tags. However, different studies have different numbers of categories for these properties. We point out that MovieShots’ four categories for shot movement do not conform to the definition in film theory. Therefore, We have consulted Daniel Arijon’s seminal work, “Grammar of the Film Language”^16^, to redefine the categories of attributes in our dataset.

In FullShots, there are eight types of shot movement: (1) *Static*, which refers to a stationary camera that remains in a fixed position and angle. (2) *Push*, which refers to the camera moving forward along a direction parallel to the object being filmed, gradually enlarging the image. (3) *Pull*, which refers to the camera moving backward along a direction parallel to the object being filmed, gradually reducing the image. (4) *Shake*, which refers to the camera shaking or trembling during motion, usually due to unstable motion or the use of handheld cameras. (5) *Pan*, which refers to the camera moving horizontally on a plane, from left to right or from right to left, usually used to capture horizontal motion. (6) *Tilt*, which refers to the camera moving vertically on a plane, from top to bottom or from bottom to top, usually used to capture vertical motion. (7) *Follow*, which refers to the camera moving along with the object being filmed, maintaining a constant relative position with the object. (8) *Boom*, which refers to the camera moving vertically, from low to high or from high to low.

**Figure 2:**
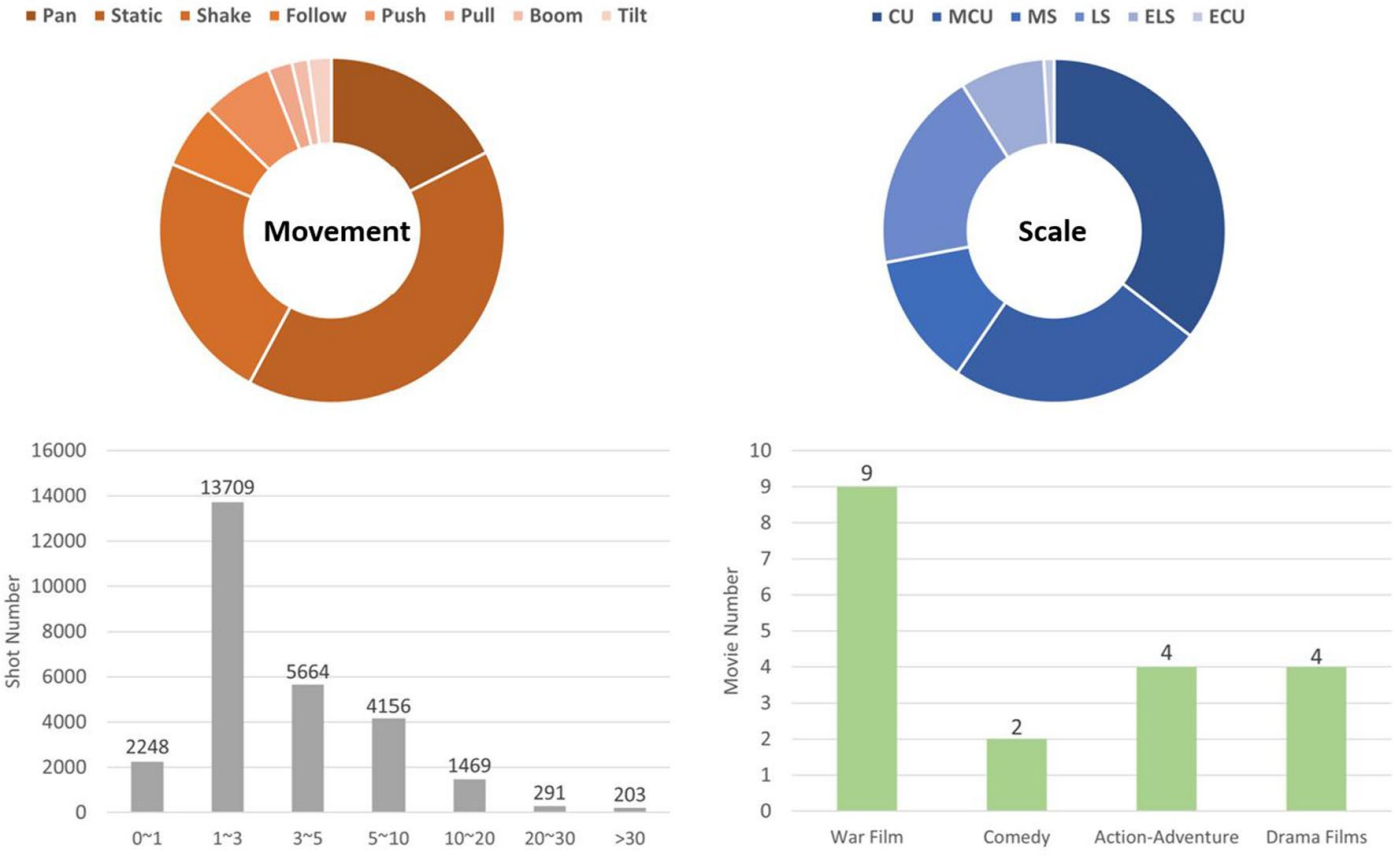
Statistics of FullShots. The pie graphs show the distribution of categories within each shot attribute. The histograms show the distribution of shot duration and the film genre statistics among 19 movies.

There are six types of shot scale: (1) *Extreme Close-Up* (ECU), which refers to the camera filming a part of an object or person from a very close distance, such as the eyes, mouth, and other parts. (2) *Close-Up* (CU), which refers to the camera filming a specific part or area of a person or object, usually the face, hands, or other parts, at a relatively close distance. (3) *Medium Close-Up* (MCU), which refers to filming a person or object from the waist or chest up to the head, at a moderate distance. (4) *Medium Shot* (MS), which refers to filming a person or object from the waist or knees up, usually including the whole body and the surrounding environment. (5) *Long Shot* (LS), which refers to filming a larger image range, including the person or object and its surroundings. (6) *Extreme Long Shot* (ELS), which refers to the widest range of camera shots, including a broad view of the environment, usually used for natural scenery, buildings, city streets, and other scenes. Figure 2 shows the percentage of categories,the distribution of shot duration and the film genre statistics in FullShots.

### Dataset construction

In order to align with the actual demands of film analysis, we propose FullShots, a dataset composed of movie segments. We use the PySceneDetect Library^8^ to produce approximately 32K shot samples for each movie, from which we manually remove ineffective shots and re-segment some with error boundaries. This results in a total of 27K valid shots that are annotated for shot movement and scale attributes by trained personnel in two rounds, with final annotations determined by a group leader. All annotators have a background in film studies.

Table 1 compares FullShots with other movie shot classification datasets in terms of the number of shot samples and source videos. Table 2 shows the comparison of MovieShots and FullShots in terms of *Train*, *Val*, and *Test* set division, and Table 3 displays the difference in shot duration distribution between MovieShots and FullShots. Compared to other shot classification datasets, FullShots has fewer source videos, as we do not impose further constraints on shot type (e.g., Subject Centric Shots in MovieShots^15^). Additionally, since the shot samples in MovieShots are from movie trailers, mainly demonstrating highlights of movies with faster editing rhythms, thus the average shot duration is shorter with more concentrated distribution. In contrast, FullShots shot samples are directly obtained through shot segmentation from original movies, resulting in a more evenly distributed range of shot durations (i.e., the number of shots exceeding 5 s is far greater than the corresponding sample count in MovieShots). We argue that the goal of shot classification should not be limited to short shot clips. Clearly, FullShots is more in line with practical shot classification needs and more diverse.

**Table 1:** Comparison of shot type classification dataset.

Dataset	Shots	Videos	Scale	Movement
Lie^39^	327	327		$\checkmark$
Cinema^40^	3000	12	$\checkmark$	
Context^19^	5054	7	$\checkmark$	
CineScale^13^	–	124	$\checkmark$	
MovieShots^15^	46,857	7858	$\checkmark$	$\checkmark$
MovieShots2^21^	15,091	282	$\checkmark$	$\checkmark$
FullShots	27,740	19	$\checkmark$	$\checkmark$

**Table 2:** Statics of *MovieShots* and *FullShots*.

Dataset	Train	Val	Test	Total
MovieShots^15^	32,720	4610	9527	46,857
FullShots	15,393	2694	9654	27,740

**Table 3:** Shots duration of *MovieShots* and *FullShots*.

Dataset	0–1	1–3	3–5	5–10	10–20	20–30	>30
MovieShots^15^	12,022	18,833	2471	821	95	13	4
FullShots	2248	13,709	5664	4156	1469	291	203

## Experiments

### Experiment configuration

We evaluate the performance of our proposed LWSRNet on both MovieShots and FullShots datasets. For MovieShots, we split the dataset into training, validation, and testing sets in a ratio of 7:1:2. For FullShots, we divided the dataset in a 9:2:8 ratio, ensuring that movie shots in the training, validation, and testing sets are from different movies.

We use the sampling method from^33^ for the input modalities, with $N=8$ for MovieShots and $N=16$ for FullShots. We train each model for 80 epochs using mini-batch SGD, with a batch size of 64 and momentum of 0.9. We use a learning rate that decreases by a factor of 10 at the 20th, 40th, and 60th epochs. The experiments are conducted on a single 4090 GPU using PyTorch. The widely used Top-1 accuracy is used as the evaluation metric.

### Overall result analysis

For MovieShots, we display the results of various methods in Table 4. For FullShots, since the code in^15^ is not publicly available, we reproduce SGNet according to the paper, and the results are shown in Table 5. Table 6 presents the parameter and computation comparisons of different methods. In particular, *frame+extra* denotes using $I_{frame}$, $I_{flow}$ for shot movement classification and $I_{frame}$, $I_{seg}$, $I_{saliency}$ for shot scale classification.

#### Results on MovieShots

We evaluate several methods on this dataset, and the overall results are presented in Table 4, with SGNet (img+flow) being the baseline method indicated by the * symbol. raditional methods such as DCR, CAMHID, and 2DMH performed poorly in both movement and scale tasks, with results differing by 20–30$\%$ from those of deep learning methods. This confirms that hand-designed are inadequate for extracting relevant information from shot samples.

Among the deep learning methods, I3D-ResNet50 (img) outperforms SGNet (img) in Acc$_{\text{M}}$ (which represent the accuracy in *movement*) by 7.1 but performs worse by 10.4 in Acc$_{\text{S}}$ (which represent the accuracy in *scale*), suggesting that 3D-CNNs are better at learning temporal features. SGNet (img+flow) shows slight improvements in Acc$_{\text{S}}$ and Acc$_{\text{M}}$ compared to TSN-ResNet50 (img+flow), which also validates the effectiveness of separating foreground and background in SGNet. The methods proposed after^15^, such as VH-Pooling and Ro-TextCNN, achieve similar or better results compared to SGNet (img+flow).

**Figure 3:**
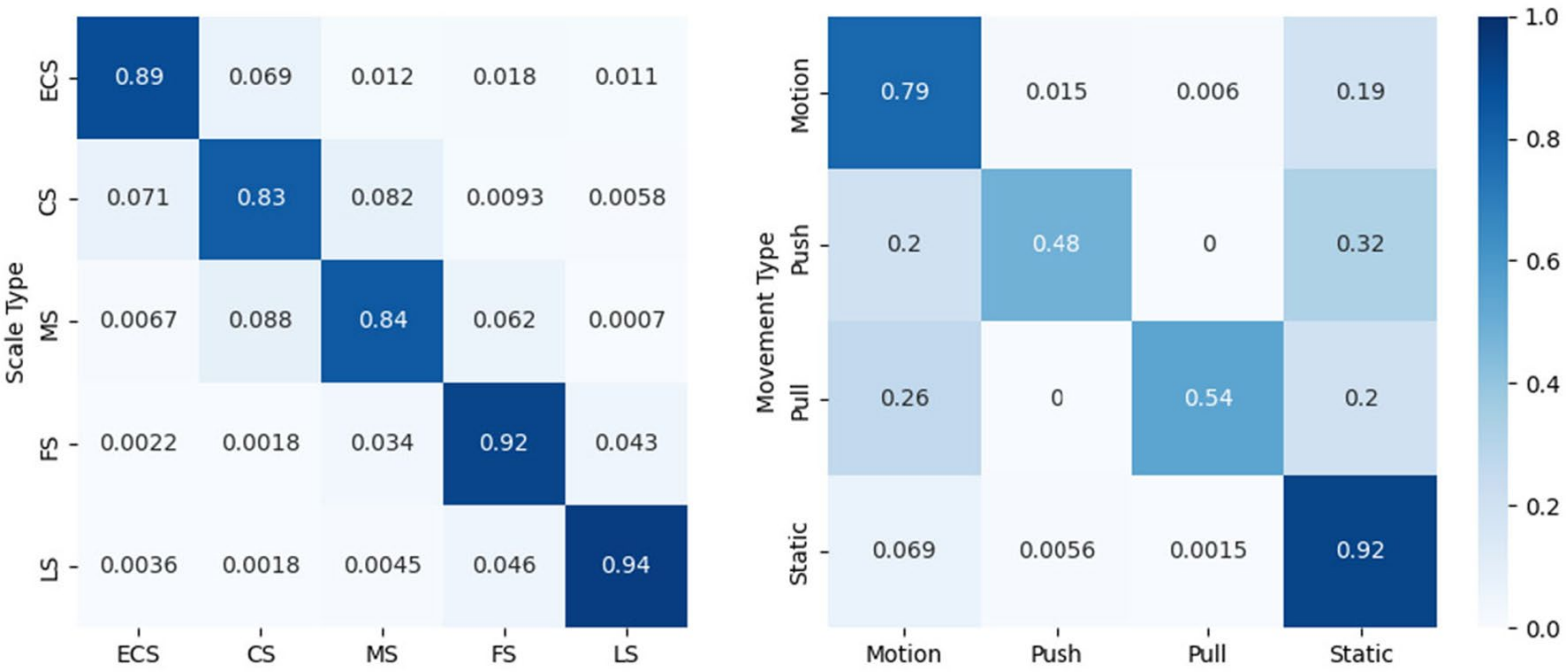
Confusion matrix of shot type classification on MovieShots Dataset^15^ by *Ours (frame+extra)*.

In our framework, Acc$_{\text{M}}$ has demonstrated significant improvement compared to previous methods. Additionally, Acc$_{\text{S}}$ is surpasses that of SGNet (img+flow) by a slight margin of 0.5. This serves as validation for the effectiveness of our proposed architecture in both tasks. Figure 3 illustrates the confusion matrix for the classification in MovieShots, in the shot movement classification task, it is worth noting that both the *Push* and *Pull* categories should be classified as *Motion* . This explanation accounts for the misclassification of certain instances within the *Push* and *Pull* classes, as observed in the results.

When using multi-modal video inputs, we observe a 0.2 boost in Acc$_{\text{S}}$ and a 4.6 boost in Acc$_{\text{M}}$. Moreover, we find that pre-trained the model used in shot scale classification improves the accuracy of small amplitudes.

**Table 4:** Overall results on MovieShots.

Models	Acc*S*	Acc*M*
DCR^29^	51.53	33.20
CAMHID^26^	52.37	40.19
2DMH^27^	52.35	40.34
I3D-ResNet50 (img)^1^	76.79	78.45
TSN-ResNet50 (img+flow)^33^	84.10	77.13
SGNet (img)^15^	87.21	71.30
SGNet (img+flow)^15^*	87.50	80.65
VH-Pooling(img)^20^	87.19	–
Ro-TextCNN (img+flow)^17^	–	82.85
Ours (frame)	87.77	81.63
Ours (frame)-pretrain	87.85	80.35
Ours (frame + extra)	87.81	**86.56**
Ours (frame + extra)-pretrain	**88.03**	86.27

Significant values are in bold.

#### Results on FullShots

The overall experimental results on FullShots are displayed in Table 5. When using $I_{frame}$ as the only input, our LWSRNet outperforms I3D(img) by a margin of 9.3 on Acc$_{\text{S}}$ and 3.8 on Acc$_{\text{M}}$. When using multi-modal input, LWSRNet improves by 0.9 in Acc$_{\text{S}}$ and 3.2 in Acc$_{\text{M}}$ compared to SGNet (img+flow). Additionally, we observe that the pre-training model does not effectively improve the model’s performance, possibly due to the mismatch in feature distribution between the source data from *Human-Centric *Kinetics400^41^ and *Broader* FullShots. These results indicate that FullShots presents a more challenging dataset than MovieShots.

**Table 5:** Overall results on FullShots.

Models	Acc*S*	Acc*M*
I3D (img)^1^	45.17	54.73
R3D (img)^42^	52.74	61.80
SGNet (img+flow)^15^*	56.32	59.95
Ours (frame)	54.47	58.50
Ours (frame)-pretrain	56.86	58.29
Ours (frame + extra)	**57.21**	**63.15**
Ours (frame + extra)-pretrain	57.19	63.03

Significant values are in bold.

#### Model efficiency

We have conducted a parameter analysis to compare the efficiency of various networks. SGNet-s represents the use of a student network for Subject Generation in SGNet, while SGNet-o represents the use of R3Net for Subject Generation in SGNet. LWSRNet-movement/scale represents our framework for movement classification and scale classification. In particular, to ensure uniform input parameters, I3D and R3D use $I_{frame}$ as input, SGNet and LWSRNet-movement use $I_{frame}, I_{flow}$ as input, and LWSRNet-scale uses $I_{frame}, I_{seg}$ as input.

As shown in Table 6, given the same number of input video modalities, LWSRNet has 48$\%$ fewer parameters and 55$\%$ fewer GFLOPs compared to SGNet-s, while still achieving better results on both datasets. This demonstrates the significant efficiency advantage of our proposed model.

**Table 6:** Parameters and computational complexity of models.

Models	Trainable params (M)	Non-trainable params (M)	Total params (M)	GFLOPs
I3D^1^	12.30	0.00	12.30	55.75
R3D^42^	33.18	0.00	33.18	22.62
SGNet-o^15^	111.83	0.00	111.83	69.48
SGNet-s^15^	74.31	0.00	74.31	49.32
LWSRNet-movement	24.24	0.00	24.24	21.97
LWSRNet-scale	27.20	11.20	38.40	22.48

### Ablation studies

To verify the effectiveness of our proposed architecture, we conduct four ablation studies: (1) the number of layers, (2) 3D-CNN backbones, (3) multi-modal video inputs, and (4) the Movement Branch and the Scale Branch.

#### Backbone layers

We test the impact of C3D backbone networks with different layer numbers on the model’s performance, as shown in Table 7. A 2-layer C3D performed significantly worse compared to a 3-layer C3D. However, the performance of a 4-layer C3D does not significantly differ from that of a 3-layer C3D. This suggests that low-level semantic features are more valuable in shot classification tasks, and the model’s operational efficiency can be enhanced by using backbone networks with fewer layers.

**Table 7:** Ablation study on backbone layers.

Layers	MovieShots	FullShots
Movement		
2	84.45	59.04
3	86.56	63.15
4	86.19	63.33
Scale		
2	83.20	51.64
3	87.81	57.21
4	87.94	57.23

#### 3D-CNN backbones

To evaluate the impact of different 3D convolutions as the backbone network on model performance, we conduct experiments while keeping the number of layers in the backbone network constant. The results, presented in Table 8, show that using C3D as the backbone network yield better performance than using R(2+1)D and R3D. However, it is difficult to explain theoretically why choosing C3D as the backbone network is better. We ultimately selected C3D as the backbone network for our architecture after conducting a series of experiments.

**Table 8:** Ablation study on 3D-CNN backbones.

Backbones	MovieShots	FullShots
Movement		
C3D^2^	86.56	63.15
R(2+1)D^43^	83.21	58.86
R3D^42^	82.12	61.22
Scale		
C3D^2^	87.81	57.21
R(2+1)D^43^	80.66	54.45
Res3D^42^	86.90	56.68

#### Multi-modal input

In this section,we analyze the influence of multi-modal video input on both shot movement classification and shot scale classification. The results are shown in Table 9. For movement type, we find that when using $I_{flow}$ as the only input modality, there is a discernible enhancement in accuracy on both the MovieShots (+ 2.89) and FullShots (+ 4.39), as opposed to utilizing $I_{frame}$. We also attempted to use all four video modalities as input features, and the results show that the model’s performance remained mostly the same, suggesting that the SE Block^37^ in the LMF Module can automatically allocate more weight to useful modalities.

For scale classification, we use [$I_{frame}$, $I_{seg}$] and [$I_{frame}$, $I_{saliency}$] as inputs to analyze two features. Results on MovieShots indicate that using $I_{seg}$ and $I_{saliency}$ as additional inputs can effectively improve the performance. However, in FullShots, using $I_{saliency}$ alone achieves higher accuracy than using both $I_{seg}$ and $I_{saliency}$ as input, suggesting that $I_{seg}$ may reduce accuracy due to the complexity of shots in FullShots. This is possibly because the segmentation maps obtained from pre-trained models may not accurately guide the information in FullShots.

**Table 9:** Ablation study on multi-modal input.

Input modalities	MovieShots	FulllShots
Movement		
Frame flow	86.56	63.15
Flow	84.52	62.89
Frame flow seg saliency	86.31	63.44
Scale		
Frame seg saliency	87.81	57.21
Frame seg	86.37	55.51
Frame saliency	87.67	57.52

#### Movement branch and scale branch

In our architecture, the Movement Branch and Scale Branch are used to supplement the output features of the backbone network with additional information. The characteristics of shot movement classification and shot scale classification are respectively considered in the design. As shown in Table 10, removing either of these branches resulted in a noticeable decrease in the model’s performance. While both tasks are related, shot scale classification focuses more on spatial features, whereas shot movement classification is more time-dependent. Therefore, incorporating a specialized module for each task beyond the backbone network significantly improves the model’s performance.

**Table 10:** Ablation study on movement branch and scale branch.

	MovieShots	FullShots
Movement branch	86.56	63.15
Without movement branch	82.12	60.67
Scale branch	87.81	57.21
Without scale branch	85.78	56.83

.

### Use of human participants

Y.L., T.L. and and four other volunteers with a background in film studies were involved in the annotation of the dataset. We claim that our study is only an annotation of the video data and does not include any form of study of human behavior.

## Conclusion

In this work, to further explore the task of Cinematographic Shot Classification, we collect a large shot dataset called FullShots, which includes 27K shots extracted from 19 movies, covering a wider range of shot types and movements than the benchmark dataset MovieShots. Moreover, we propose LWSRNet, a Lightweight Weak Semantic Framework for Cinematographic Shot Classification, which can effectively extract temporal and spatial features from multi-modal inputs. Our experimental results indicate that LWSRNet outperforms other methods on both FullShots and MovieShots datasets while having fewer parameters and computations. Overall, this work provides a significant contribution to the field of cinematography analysis, improving the accuracy of shot classification and offering a valuable dataset for future research.

## Data Availability

*MovieShots* dataset analysed during the current study can be available in https://movienet.github.io/projects/eccv20shot.html. Due to strict copyright restrictions on films, we are unable to share any video data in any way from *FullShots* dataset , but parts of label files are available from the corresponding author on reasonable request.
